# Auscultation and Percussion

**Published:** 1855-04

**Authors:** 


ART. III.—*Auscultation and Percussion.* By Dr. JOSEPH SKODA. Translated from the fourth edition by W. O. MARKHAM, M. D., Assistant Physician to St. Mary's Hospital. Philadelphia. Lindsay & Blakiston. 1855. 8vo., pp. 380.

(*Continued from March number.*)

By vesicular breathing SKODA understands the sound heard in inspiration alone—and allows no relation between it and the expiratory murmur. He does not differ in opinion from LAENNEC as to the cause of its production. It may be unattended by expiratory sound; when this is present, it always indicates the existence in the bronchial tubes of some obstruction to the egress of the air from the lungs.

Many, perhaps the majority of auscultators, set down the relation of the expiratory to the inspiratory murmurs as one to three, or two to five. SKODA adopts FOURNET'S figures which make the duration of the former only one-fifth of that of the latter.

Dr. WALSHE and SKODA are the only very distinguished authorities in physical diagnosis who affirm that the prolonged expiratory murmur with increase of its intensity is not an important diagnostic sign. Skoda regards it, unless it has a bronchial or some other character than that proper to it, as simply indicating that the air in passing out of the lungs meets with some obstruction in the bronchial tubes.

Fournet, Barth and Roger, Stokes, Bennett, Thompson, our countryman, Dr. Jackson, and a number of other physicians who have attained just celebrity as auscultators, esteem the prolonged expiratory murmur, when heard in a particular locality, as one of the earliest as it is one of the most positive signs of pulmonary tuberculosis We ourselves have always so regarded it, and believe that our estimate of its value has been the legitimate result of our practical experience.

Under the term râles our author includes only such sounds as resemble the breaking of bubbles in water, or the crackling as of burning wood. He divides the râles as he does the voice and respiration, so far only as in his opinion the division possesses practical value. He recognises five, as follows:—

"Vesicular râle; consonating râle; dry crepitating râle, with large bubbles; indeterminate râles and râles accompanied by amphoric resonance and metallic tinkling."

Skoda agrees with Laennec and Andral as to the manner in which and the place where the vesicular râle is produced. The consonating râle cannot occur unless the conditions necessary for the production of consonance are present. Its significance is hence the same as bronchophony and bronchial breathing; generally speaking, it indicates, according to our author, the presence either of pneumonia or tubercular infiltration, being seldom observed in pleuritic effusion. Indeterminate râles are those which are neither vesicular nor consonating, and are not accompanied by amphoric resonance or metallic tinkling; as their name sufficiently indicates they are of no diagnostic value. Skoda believing that the snoring, whistling, and hissing sounds may arise when the lung-tissue is normal, or under any of its abnormal states, asserts that they afford no information as to its condition, except when they are consonant. He says they consonate like the voice, the respiration and the râles, and when this is the case they are accompanied by a resonance resembling that of the bronchial voice and indicate the same condition of lung-tissue.

Amphoric echo and metallic tinkling are not observed unless there is some large cavity present, the walls of which are capable of reflecting sound. LAENNEC thought these sounds were not produced unless both fluid and air were present in the cavity. SKODA does not regard the presence of fluid as at all necessary. Of metallic tinkling he says:—"In addition to its being heard as a resonance of the voice, of the breathing, and of whistling sounds, it may present itself in large cavities, as the resonance of a râle, situated in a distant, but communicating bronchial tube, or as a resonance of a râle which has its origin at the mouth of the opening into the cavity, or when many cavities communicate together, at the opening of communication between them—where, in fact, the air may pass in and out during respiration, without being obstructed by fluid—or as the resonance of a râle produced in cavities, through violent concussion of their fluid contents, by coughing, &c."

The cough, according to SKODA, does not afford any signs different from those already described.

LAENNEC ascribes the friction sound in most cases to superficial interlobular emphysema; and also supposed that it would manifest itself when any cartilaginous, tubercular, or other hard tumors, projected above the surface of the lung. SKODA holds the reverse of this opinion to be true and adopts the theory of Dr. REYNAUD which attributes the friction sound to the pleural surfaces being roughened.

The auscultatory phenomena of the *organs of circulation* are next considered. As among the causes of the *impulse* of the heart SKODA adopts the recoil theory of GUTBROD:—"It is a well known physical law, that when a fluid escapes from a vessel, the equality of pressure produced by the fluid on the walls of the vessel is lost, for there is no pressure at the opening whence the fluid escapes: but at that part of the vessel which is opposite to the opening, the pressure is still exerted. This pressure it is which set Segner's wheel in motion, and produces the recoil of fire-arms, etc. By contraction of the ventricles, the pressure which the blood exerts upon the walls of the heart, opposite to the opening whence the stream escapes, causes a movement of the heart in a direction contrary to that of the stream of blood, and by this movement the impulse of the heart against the walls of the thorax is produced.

The heart is driven in a direction contrary to that of the arteries, with a force proportionate to the quantity and the velocity of the current of the blood."

Among the other causes which assist in the production of the heart's impulse, may be mentioned the lengthening of the pulmonary artery and aorta during the systole as especially worthy of notice, and also the change of form and rigidity the heart assumes during this action of its fibres. These several different causes are not always found associated together in the production of the heart's impulse; "one only may be in operation, or there may be several, the force of the impulse is necessarily connected with the rapidity and completeness of the contractions, and with the size of the organ itself." The force of the impulse is also affected by the size of the arterial opening, and the quantity of blood contained in the ventricle, though there is no question that the impulse of the heart may be caused by mere change of its form, independent of any expulsion of blood from its ventricles.

As regards the cause of the *sounds* of the heart Skoda thinks the first sound is for the most part the result of the sudden stoppage of the flow of blood towards the auricles consequent upon the distension of the mitral and tricuspid valves. The state of tension in which the valves are suddenly thrown by the presence of the blood, contributes in some degree to the production of the first sound. It is also "produced by the impulse of the heart against the walls of the thorax. A blow struck with the finger, or with the apex of the heart firmly compressed, against the inner surface of the thoracic walls, produces a chinking (*klirrend*) sound, which differs in no particular from the ordinary first sound of the heart. If a part of the walls of the heart be somewhat separated from the thoracic walls during the ventricular diastole, but strike against them during the systole; or even if the heart beat against some other part of the thoracic walls during its systole than that beneath which it lay during the diastole, still, in either case, a chinking sound, or one exactly resembling the ordinary first sound of the heart, will be produced; for the heart's substance becomes firm during its systole." With all these causes of the first sound however, Skoda admits that in practice we meet with many cases in which its explanation in unsatisfactory.

"The explanation of the causes of the *second* sound presents still greater difficulties than the first; we cannot affirm that the second sound is always formed in the ventricles, when the condition of the heart is normal; for it seems probable, and sometimes indeed certain, that the second sound heard over the heart arises in the arteries, and, on account of its intensity, is propagated to a distance. But there are cases in which we are forced to admit that the second sound arises in the neighborhood of the ventricles; cases, for instance, in which the second sound is barely audible over the base of the heart, but very loud and clear over its apex. A second sound of this description cannot be explained by the impulse of the heart against the thoracic walls, for there is no impulse during the ventricular diastole. It is possible that the second sound may be occasionally produced by the impulse of the blood against the walls of the ventricles during the diastole; such an impulse undoubtedly takes place in the left ventricle, when the aortic and mitral valves are defective."

The murmurs of the heart may arise either within the cavities of the heart, or in the arteries or their coats, or within the pericardium. It is unnecessary to designate the terms by which these murmurs are known as they occur in the different localities. LAENNEC, it will be remembered, believed that endocardial murmurs were not connected with any structural changes of the heart, but were wholly the products of spasm. Later observers, however, have established that in many instances organic changes of the heart's structure are associated with these sounds, and changes of such a nature as to render their causes very explicable. The general opinion, according to SKODA, now is, that endocardial murmurs are caused by friction of the blood against the walls of the heart, or against its valves, and may depend on organic change or be wholly independent of it. Endocardial murmurs which cannot be directly referred to structural changes of the heart, undoubtedly depend, says SKODA, for the most part, upon friction between the blood and the walls of the heart. But how this friction produces the murmurs, he confesses himself unable to explain. The fact that murmurs may also be produced within the heart's cavities, by the rapid flow of a small stream of blood against blood that is quiescent, or moving less rapidly, or in an opposite direction to it, he considers established. The opinion that endocardial murmurs are caused by a particular condition of the blood Skoda regards as hypothetical, and says he must continue to do so until the particular condition of the blood on which it depends has been demonstrated. While he admits that murmurs are sometimes observed in the heart after great sanguine losses, and in anæmia, he thinks the occurrence so rare as not to warrant the watery condition of the blood being considered as the only cause of the murmurs. Andral believes that he has heard endocardial murmurs in cases of simple general plethora, and has attempted to explain the fact by supposing that the cavities are over small, relatively to the quantity of blood which should pass through them in a given time. Skoda has never observed a murmur that was thus produced and asserts that the flow of blood through the cavities of the heart does not depend on the blood, but upon the action of the heart.

The division of endocardial sounds into blowing, rasping, etc., on which so much stress has b en laid by Hope and most of the French auscultators he thinks altogether unnecessary:—"It is altogether in vain," he remarks, "to attempt to distinguish too particularly between the different kind of murmurs; indeed it matters little, as respects any conclusions which may be deduced from our observations, whether the murmurs are of a blowing, sawing, or rasping character. We often find a bellows murmur suddenly converted into a sawing or rasping murmur, through accidental increase of the heart's action. Murmurs arising within the cavities of the heart, and the arterial murmurs, not only become louder when the heart's action is increased, but they also acquire a rougher, sharper, and more acute character; the reverse of this happens when the heart's action is weakened, the murmur becoming muffled and very indistinct or disappearing altogether." He regards the seat of the murmur and the time of its occurrence whether following the tic or the tac, as of far greater importance.

Defect of the aortic, tricuspid, and mitral valves, constriction of the mitral orifice, or of the mouth of the aorta, and irregularities, such as excrescences, concretions and blood coagula, are the organic changes of the heart which produce endocardial murmurs.

"Cardiac murmurs, unassociated with appreciable alteration of the heart's structure, are observed in the course of many different diseases. Chlorosis may be particularly instanced; but here, the murmers are much more frequently heard in the arteries than in the heart. The same remark applies to the faulty state of the blood produced by the cachexia of cancer.

"A very loud blowing murmur has been sometimes heard, during the systole of the heart, in cases of acute rheumatism, where no change of the heart's structure could be found after death. During pregnancy, in puerperal diseases, at the onset of typhus, of small pox, and of severe inflammatory diseases, and under many other circumstances, a systolic murmur (either replacing the sound, or accompanying it) is occasionally heard in the heart or in the arteries."

Up to this point even though we have not been altogether satisfied with the explanation offered by SKODA of many auscultatory phenomena and much less with the diagnostic value which he has ascribed to one or two of them, we have not ceased to admire and commend in every instance his efforts to simplify the subject. We cannot here, however, avoid the remark that in our opinion he has by no means given to the murmurs which are independent of any structural change of the valves, and, if we may be allowed the expression, inorganic, and arise in the course of other diseases,—the consideration which they deserve.

There is no kind of murmur which is heard within the left ventricle, that may not also be heard in the aorta. The blowing murmur heard in the subclavian and carotid arteries of perfectly healthy subjects, and that observed in the thyroid arteries in cases of bronchocele. and the same sound heard in other arteries of the body, are not of very easy explanation. It is difficult, says SKODA, to determine whether they are caused by friction of the blood against the walls of the arteries, or by vibrations of the walls excited by their distension.

*Pericardial* murmurs were not known to LAENNEC. It is unnecessary to repeat here the division which has since been made of them. The credit of having first directed attention to the diagnostic importance of these sounds is due to COLLINS, though the profession generally did not become familiar with either their names or signification until the appearence of Stokes' paper on pericarditis.

Bouillaud, Graves, Walshe, and Stokes, believe the distinction between pericardial and endocardial murmurs a very easy matter in most instances, while Skoda declares it is exceedingly difficult and often even impossible. He says that except endocardial murmurs correspond to the rhythm and the natural sounds of the heart which every one knows they frequently do not do, whilst pericardial sounds follow the movements of the heart, he knows of no sign by which they can be distinguished from each other. The greater extent over which pericardial murmurs may be heard, their greater diffusion and superficiality he does not regard as sufficient to enable the observer to distinguish them from murmurs produced within the heart. According to his experience there is no kind of endocardial murmur except the whistling, which may not be imitated by a pericardial sound; and no pericardial murmur which may not be imitated by one originating in the endocardium. He does not believe the pericardium capable of producing a murmur if its surface be smooth and uncovered by plastic exudation.

"A friction sound accompanying the movements of the heart, does not invariably arise from the inner surface of the pericardium; it may depend also upon a roughened condition of that portion of the pleura which covers the unattached parts of the pericardium. The sound is produced by the rubbing of the pleura which covers this free portion of the pericardium, either against the thoracic walls, or against the surface of the lungs; being caused by the action of the heart, it coincides with its movements as completely as though it had been produced within the pericardium. The murmur thus arising external to the pericardium exactly resembles the murmur arising within it. I once thought, that a distinction between them might be found in the circumstance of the external murmur being alternately increased and diminished in force during the respiration: but it appears that the internal pericardial murmur is likewise frequently affected in a similar way."

Although Skoda thinks there can be no doubt that the *bruit de diable* (the top murmur, the Nun's murmur) originates in the jugular veins, its mode of production he does not regard as very clear. He has for many years looked on it as indicating neither a deficiency of blood nor a watery condition of that fluid. HAMERNJK's explanation of the murmur is this: "the less the quantity of blood present in the vena cava, the more rapid will be the flow of blood through the jugular veins during inspiration, and the smaller the current of blood. Now, the internal jugular vein is so attached at its lower part, as of necessity always to retain a certain width: the diminished stream of blood can only fill this wide space, by passing through it with an eddying movement; this movement is communicated to the walls of the vein and the parts around, and is rendered sensible to the touch by vibrations, and to the ear by murmurs. It is well known, that the bruit de diable is only heard in the veins of the neck, and more frequently on the right side than on the left. This fact, in Hamernjk's opinion, confirms his views; for the conditions which, according to him, are necessary for the production of the bruit, exist only in the internal jugular veins, and in a much more perfect manner in the right than in the left. The bruit has hitherto been considered a sign both of anæmia and of spanæmia; Hamernjk looks upon it as a sign of anæmia only."

We cannot overrate the importance of being able to determine whether the murmurs heard arise in the heart and arteries, or in the pericardium; for the murmurs, as we have elsewhere remarked, which originate in the pericardium, may be heard over the same regions as those which originate in the heart and arteries. Our limits forbid, even if it were not unnecessary, our giving the differential diagnosis of these several murmurs. And then the points laid down by SKODA do not materially differ from those to be found in other authors. The same remark applies to the signification attached by SKODA to the various endocardial murmurs.

Part second is occupied with a description of the phenomena obtainable by the aid of auscultation and percussion appertaining to special conditions of the thorax and abdomen. We find no peculiar views relative to any subject in this portion until we come to the physical diagnosis of pneumonia. Here the Viennese teacher declares that the auscultatory and percussion signs are the same whether the hepatization be red or grey, whether the lung be hard or soft, of firm or loose texture. We need not stop to say how much this statement is opposed to the orthodox faith on this subject. Even with his unrivaled powers of diagnosis, Skoda says he has never in any single instance, recognized the presence of pneumonic abscess by the aid of ausculta-ion or percussion. He has repeatedly made post mortem examinations of patients suffering from pneumonia in whose lungs newly formed abscesses were found, and, in every case, the abscesses though communicating with the bronchial tubes, were filled with pus or sanies. According to his belief, there are no physical signs by which we can discriminate between inflamed lung when it contains a small quantity of air, and when it contains no air at all.

Laennec thought the impulse, synchronous with the beats of the heart, which is often observed over hepatized lung, was produced by the mere propagation of the heart's impulse; but Skoda says there is no doubt whatever, that the impulse felt over either a hepatized lung or a lung infiltrated with tubercle, is caused by the pulsation of the arteries passing into the lung. Skoda rejects the opinion of Dr. Stokes respecting the nature of red hepatization, and of pulmonary abscess. The lively red color of the lungs he agrees with Rokitansky in considering as the result of anæmia; and while he admits that strong vesicular respiration occasionally precedes crepitation he says it is so very variable and inconstant, being even more so than crepitation, that it is almost worthless as a symtomp. Nor does he coincide with Dr. Stokes relative to bronchial breathing, nor the statement that red hepatization is indicated by the simultaneous appearance of bronchial respiration and of a peculiar sharp muco-crepitant râle. The following views are in many respects so diametrically opposed to the teachings of most auscultators that we cannot forbear quoting them entire:—

"In the great majority of cases, according to my experience, the auscultatory signs of pneumonia do not follow in the order assigned to them by Laennec.

"It frequently happens that the fine equal-bubbling râle is not heard at the commencement of pneumonia; we meet with the unequal bubbling râle—Laennec's mucous râle—or whistling and sonorous sounds, much more often at this period. In some rarer cases, the disease at its onset is unaccompanied by any râle at all; the inflamed parts yielding an indeterminate, or vesicular, or very noisy respiratory murmur, which passes at last into bronchial respiration.

"So long as the hepatization continues, we may have bronchial breathing unmingled with râles, or combined with consonating or non-consonating hissing, and sonorous sounds, or again the breathing may be indeterminate, and with or without râles or hissing sounds, &c.; or there may be no respiratory murmur, nor any râles whatever, audible. The consonating râles may be formed of smallish bubbles, and when of a dry character resemble crepitation; or the term crepitation might rather, in many instances, be applied to the consonating râle. It would be hard to say, whether the râle which is heard previous to the hepatization, or the consonating râle attendant upon complete hepatization, has been most frequently taken for Laennec's crepitating râle.

"The period of resolution of pneumonia does not invariably commence with the appearance of the crepitating râle; in most cases it is attended by a great variety of râles, or by whistling and sonorous sounds. In some rare cases, no râles whatever appear during this stage of the inflammation; the bronchial breathing being at first indeterminate, and at last vesicular.

"The crepitating râle, or a râle resembling it, is heard during the resolution of moderately severe cases of pneumonia; it is also occasionally observed in severer cases, at a more advanced period of the resolution, when the secretion has become scanty. In the greater number of cases, the vesicular breathing does not return immediately upon the resolution of the disease; but we generally find, after all the functions are restored to their healthy condition, and percussion no longer yields any abnormal sound, that the respiration still remains indeterminate, or that râles, or hissing, whistling, and sonorous sounds continue. Auscultation yields the same signs when the resolution is incomplete.

"It follows from the above, that the presence of pneumonia cannot be determined by the auscultatory signs alone, and that these are often very indefinite, and that bronchophony, bronchial breathing, and other consonating sounds, as well as vesicular breathing, and fine equal-bubbling râles, are signs, which of themselves do not enable us to draw accurate conclusions as to the condition of the lung-parenchyma; in forming our diagnosis, we must also take into consideration every other symptom attainable by percussion, and by other means at our command."

The succeeding chapters are occupied with the diagnosis of the various diseases found in the pulmonary and circulatory apparatus, and abound in the most interesting descriptions. We have endeavored in this notice, which is already much longer than we proposed when we undertook it, to confine ourselves mainly to those portions of Skoda's work wherein he held doctrines opposed to those contained in other authors on the same subject. It is our deliberate opinion that since the great work of Laennec we have had none equal to Skoda's. In originality of thought, closeness of reasoning, condensation of style, and clearness of conception and expression, it is infinitely superior to the many ephemeral productions which load our table.

